# Function and Dynamics of Tetraspanins during Antigen Recognition and Immunological Synapse Formation

**DOI:** 10.3389/fimmu.2015.00653

**Published:** 2016-01-11

**Authors:** Vera Rocha-Perugini, Francisco Sánchez-Madrid, Gloria Martínez del Hoyo

**Affiliations:** ^1^Servicio de Inmunología, Instituto de Investigación Sanitaria La Princesa, Hospital de la Princesa, Madrid, Spain; ^2^Vascular Pathophysiology Area, Centro Nacional de Investigaciones Cardiovasculares Carlos III (CNIC), Madrid, Spain

**Keywords:** tetraspanins, tetraspanin-enriched microdomains, adhesion receptors, immunological synapse, T-cell activation

## Abstract

Tetraspanin-enriched microdomains (TEMs) are specialized membrane platforms driven by protein–protein interactions that integrate membrane receptors and adhesion molecules. Tetraspanins participate in antigen recognition and presentation by antigen-­presenting cells (APCs) through the organization of pattern-recognition receptors (PRRs) and their downstream-induced signaling, as well as the regulation of MHC-II–peptide trafficking. T lymphocyte activation is triggered upon specific recognition of antigens present on the APC surface during immunological synapse (IS) formation. This dynamic process is characterized by a defined spatial organization involving the compartmentalization of receptors and adhesion molecules in specialized membrane domains that are connected to the underlying cytoskeleton and signaling molecules. Tetraspanins contribute to the spatial organization and maturation of the IS by controlling receptor clustering and local accumulation of adhesion receptors and integrins, their downstream signaling, and linkage to the actin cytoskeleton. This review offers a perspective on the important role of TEMs in the regulation of antigen recognition and presentation and in the dynamics of IS architectural organization.

## Tetraspanin-Enriched Microdomains

Tetraspanins comprise a family of small proteins with four transmembrane domains and are present on the plasma membrane and intracellular vesicles of virtually all mammalian cells. The tetraspanins CD9, CD63, CD81, CD82, and CD151 have a broad tissue distribution, whereas others are restricted to particular tissues, such as TSSC6, CD37, and CD53, in hematopoietic cells (1). Tetraspanins have small and large extracellular loops (SEL and LEL, respectively) and short N- and C-terminal intracellular tails (2). The LEL domain mediates specific protein–protein interactions with laterally associated proteins and a few known ligands, while the cytoplasmic regions provide links to cytoskeletal and signaling molecules (3). Tetraspanins organize a type of cell surface membrane microdomain, known as tetraspanin-enriched microdomains (TEMs) (2, 4), based on their exceptional ability to form multimolecular complexes. Studies using novel advanced microscopy techniques in the intact membranes of living cells have provided a more complete picture of the supramolecular organization of these microdomains (5). The diversity of TEM composition is reflected by different interaction levels, in which each tetraspanin recruits one or more partner proteins forming direct and stable primary complexes, which are assembled through tetraspanin–tetraspanin interactions to form larger complexes that can vary depending on the cell type (6). However, this classical view of TEMs has recently been challenged. Super-resolution microscopy has shown that, in B cells and dendritic cells (DCs), CD53 and CD37 single clusters overlap only to a minor extent with CD81 or CD82 clusters. Moreover, CD53 and CD81 nanoclusters are in closer proximity to their partners MHC class II (MHC-II) and CD19 than to other tetraspanins (7). Additional research using super-resolution microscopy is necessary to dissect the spatial and temporal organization of TEMs in different systems.

In the context of the immune system, TEMs regulate important processes including antigen (Ag) recognition and presentation, protein trafficking, cell proliferation, and leukocyte extravasation (1). All cells of the immune system express tetraspanins, although the tetraspanin repertoire differs between cell types (3). Several receptors responsible for immune cell functions, like the Ag receptors T-cell receptor (TCR) and B-cell receptor (BCR), pathogen receptors, and MHC molecules, are included in TEMs; furthermore, both ubiquitously expressed tetraspanins such as CD81 and immune cell-specific tetraspanins such as CD37 have been shown to be important for immunity (1). In human T lymphocytes, tetraspanins CD9, CD53, CD81, and CD82 act as costimulatory molecules (8–13), and this activity is independent of the classic CD28 costimulatory pathway (12–16). T cells from mice lacking tetraspanins CD81, CD151, CD37, or Tssc6 are hyperproliferative (17–20), and CD37- and CD81-deficient mice have impaired T-cell-dependent immune responses (17, 21–23). Moreover, CD81 expression in both T and B cells is essential for T-cell activation and proper Th2 responses (24–26).

Tetraspanins are also involved in the process of leukocyte extravasation. CD81 controls integrin α4β1 avidity, being essential for monocyte and B cell adhesion under shear flow (27), and CD9 regulates LFA-1-mediated T cell adhesion under flow conditions (28). Moreover, monocyte and T cell transmigration across brain endothelial cell monolayers is significantly reduced by monoclonal antibodies against CD81 in rodent and human *in vitro* models (29). This inhibitory effect was driven by CD81 expressed in both leukocytes and endothelial cells (29). Transmigrated eosinophils exhibit reduced CD9 expression levels, and their adhesion properties are inhibited by antibodies against CD9 (30, 31). In endothelial cells, various adhesion receptors are included in preassembled tetraspanin-based endothelial adhesive platforms; these platforms coalesce at docking structures for adherent leukocytes during the transmigration process (32, 33).

Immune cells, such as T cells, B cells, and DCs, can release extracellular vesicles that are an important vehicle for intercellular communication and have a role in the regulation of the immune response by different mechanisms (34). Tetraspanins, especially CD9, CD63, and CD81, are highly enriched in extracellular vesicles and have been widely used as exosomal markers. Importantly, growing evidence suggests a functional role for tetraspanins in the biogenesis, targeting, and function of extracellular vesicles (35). In particular, high throughput quantitative proteomic approaches have demonstrated that exosomes from CD81^−/−^ mouse T lymphoblasts show an impaired inclusion of CD81 partners, including MHC molecules, BCR, ICAM-1, and Rac (36).

Together, all these observations indicate that tetraspanins influence many aspects of cellular immunity, sometimes exerting antagonistic roles, and may provide a means of manipulating the immune response for potential therapeutic applications.

## The Immunological Relevance of Tetraspanins in Antigen-Presenting Cells

### TEMs and Antigen Recognition: Interaction with Pattern-Recognition Receptors

**Figure 1:**
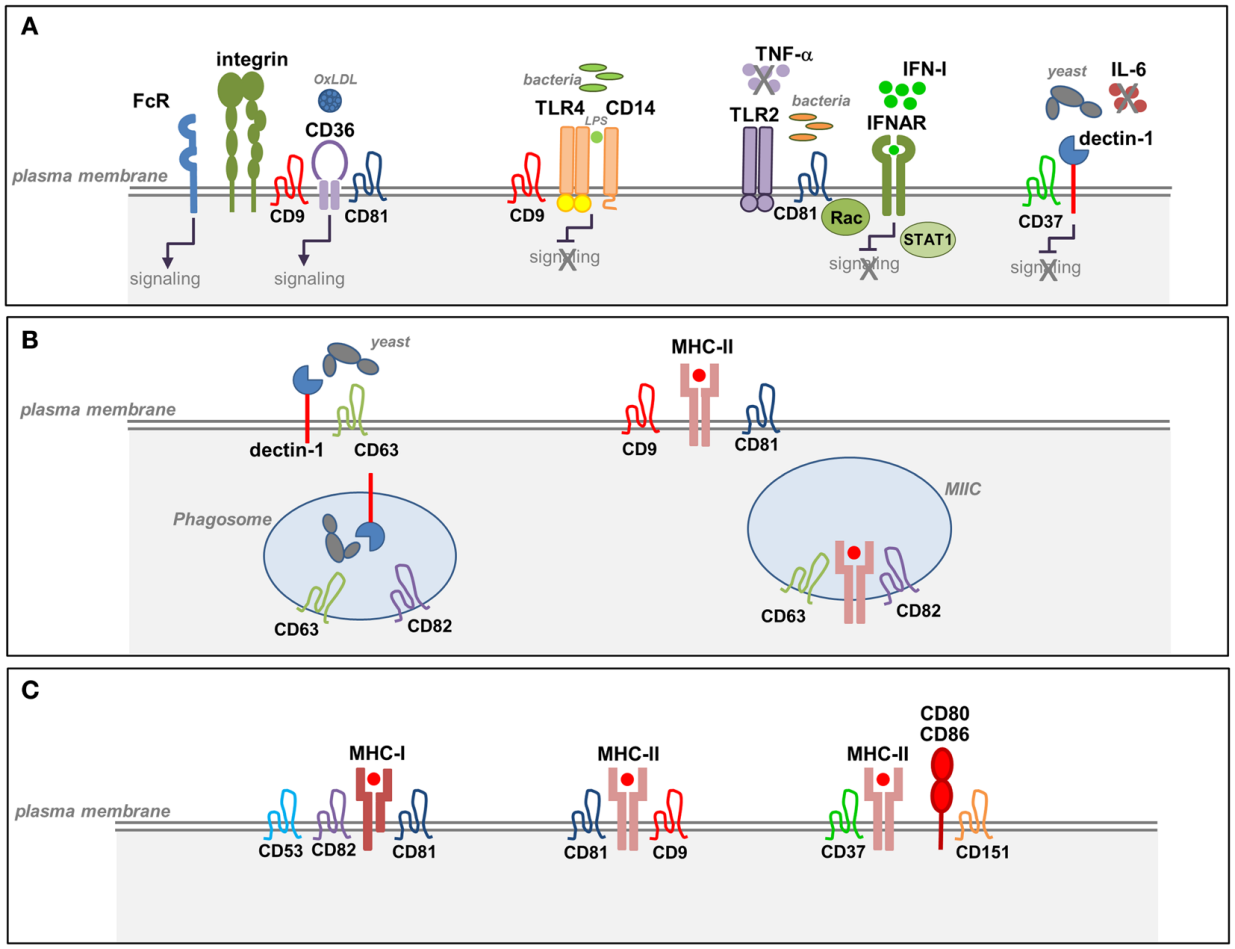
**Tetraspanins in the function of APCs**. **(A)** Tetraspanin interactions with pathogen-recognition receptors (PRRs) in APCs. Tetraspanins interact with specific PRRs at the plasma membrane of macrophages and DCs. CD37 associates with dectin-1 and inhibits dectin-1 mediated IL-6 production triggered by the recognition of fungal cell walls. CD9 forms a complex with CD14 and TLR4 and negatively regulates TLR4 signaling in response to LPS. CD81–Rac interaction inhibits TLR2- and IFNAR-signaling pathways and prevents the subsequent activation of STAT1 in response to *Listeria monocytogenes*. CD36 associates with β1 and β2 integrins and tetraspanins CD9 and CD81 forming a complex that facilitates CD36-signaling and its interaction with FcγRs. Interaction between CD9 and FcγRs promotes phagocytosis and macrophage activation. **(B)** Tetraspanin interactions during Ag processing and MHC-II biosynthesis. CD63 interacts with dectin-1 in immature DCs and promotes yeast phagocytosis. Both CD63 and CD82 are selectively recruited to yeast-containing phagosomes. CD82 and CD63 are highly enriched in MIIC compartments that contain newly synthesized MHC-II and accessory proteins. **(C)** Tetraspanin interactions during Ag presentation. Several tetraspanins associate with MHC-I and MHC-II molecules on APCs. Tetraspanins CD9, CD53, CD81, and CD37 associate with MHC-II molecules preferentially at the plasma membrane. MHC-II molecules loaded with a restricted antigenic peptide repertoire are included in TEMs together with accessory molecules and costimulatory molecules. CD9 facilitates MHC-II clustering, and CD151 is involved in the clustering of costimulatory molecules.

The plasma membrane of antigen-presenting cells (APCs) contains specialized membrane microdomains that organize the spatial distribution of MHC and associated proteins, pattern-recognition receptors (PRRs), and integrins, which are essential for efficient Ag recognition, presentation, and ultimately the activation of the T cell. APCs express a broad repertoire of specific receptors involved in the recognition and uptake of Ags from pathogens, damaged tissues, or tumor cells. In particular, pathogen-derived Ags are recognized by different PRRs that bind to conserved microbial structures called pathogen-associated molecular patterns (PAMPs) (37). The recent identification of specific PRR interactions with tetraspanins has provided new insights into the organization of Ag receptors at the APC membrane and their subsequent downstream signaling (38). In this part, we will revise the recent data that have demonstrated tetraspanin interactions with different receptors involved in Ag recognition (Figure 1).

Dectin-1 is a C-type lectin receptor (CLR) that recognizes β-glucans in fungal cell walls, triggering phagocytosis, and the antifungal immune response. Dectin-1 signaling is only activated by particulate β-glucans, which cluster the receptor in synapse-like structures from which regulatory tyrosine phosphatases are excluded (39). Two independent studies have demonstrated that CD63 and CD37 interact with dectin-1 on the APC membrane (Figure 1A; Table 1). CD63 associates with dectin-1 in immature DCs and promotes yeast phagocytosis (40) (Table 1). CD37 stabilizes dectin-1 at the APC surface, and this interaction has functional consequences since CD37 inhibits dectin-1-mediated IL-6 production in response to zymosan cell wall preparations (41) (Table 1). Moreover, CD37^−/−^ mice are protected against systemic infection with *Candida albicans* (42). CD63 has also been reported to be selectively recruited to yeast-containing phagosomes (43) (Table 1), and this observation was subsequently extended to CD82 (44) (Figure 1B; Table 1). After pathogen uptake, CD82 is rapidly recruited to the membrane of nascent pathogen-containing phagosomes prior to fusion with lysosomes (44) (Figure 1B).

**Table 1:** **Tetraspanin associations in pathogen-recognition receptors and APC functions**.

Tetraspanin	Interacting molecule	Cell type	Function	Reference
CD9	TLR4–CD14 complex	Macrophages	Regulates LPS-induced signaling	(45)
	CD36	Macrophages	Mediates CD36–integrin complex formation and ligand-bound internalization and signaling	(46, 47)
	FcγR	Macrophages	Interacts with and regulates FcγR-mediated immune responses	(47, 48)
	FcϵRI	Monocytes and DCs	Association at the membrane	(49)
	MHC-II	DCs	Association at the membrane	(50–52)
CD81	Rac1	DCs	Controls TLR2- and IFNAR-mediated bacterial recognition	(53)
	CD36	Macrophages	Regulates CD36–integrin complex formation, ligand-bound internalization and signaling	(46, 47)
	FcϵRI	Monocytes and DCs	Association at the membrane	(49)
	BCR	B cells	Controls CD19 surface expression and BCR complex downstream signaling	(3, 54)
	MHC-II	DCs	Association at the membrane	(50, 51)
CD37	Dectin-1	Macrophages	Controls dectin-1 stabilization at the membrane and signaling triggered by dectin-1 recognition of yeast cell walls	(41)
	MHC-II	B cells and DCs	Associates with and regulates MHC-II-dependent antigen presentation	(55, 56)
CD63	Dectin-1	DCs	Associates with dectin-1 and regulates yeast phagocytosis	(40, 43)
	MHC-II	DCs	Associates with peptide-loaded MHC-II and controls its surface expression	(50, 51, 57, 58)
CD82	MHC-II	Macrophages and DCs	Association at MHC-II-enriched compartments, and fungal and bacterial phagosomes	(44, 50, 51, 57)
CD53	MHC-II	B cells, DCs	Association at the membrane	(50, 55)
CD151	CD80, CD86	DCs	Regulates costimulation during Ag presentation	(56)

In addition to the reported recruitment of TLR2 and TLR4 to lipid rafts (59–61), other studies demonstrate that TLR4 associates with TEMs. In macrophages, CD9 partly colocalizes with CD14 regulating its expression, its association with TLR4, and the formation of the CD14–TLR4 complex necessary for LPS-induced signaling (45) (Figure 1A; Table 1). Using the *Listeria monocytogenes* infection model, we recently demonstrated that CD81 is able to interfere with TLR2- and interferon-α/β receptor (IFNAR)-mediated bacterial recognition in DCs, modulating the subsequent CD8^+^ T cell response (53) (Figure 1A; Table 1). Importantly, CD81^−/−^ mice are protected against lethal systemic *Listeria* infection. CD81^−/−^ DCs show increased production of proinflammatory mediators and a more efficient activation of protective cytotoxic T cells. This effect is mediated specifically through direct interaction between CD81 and Rac. Indeed, inhibition of CD81–Rac interaction in wild-type DCs using CD81 C-terminal peptides, which block CD81-mediated signaling (62), promotes the same phenotype observed in CD81^−/−^ DCs (53).

In macrophages, CD9 interacts with CD36, a scavenger receptor involved in the recognition of microbes or self-ligands, regulating CD36-mediated uptake of oxidized low-density lipoproteins (46) (Figure 1A; Table 1). CD36 clustering is necessary for the initiation of signal transduction and internalization of receptor–ligand complexes. CD36 was recently shown to form a heteromeric complex containing β1 and β2 integrins and the tetraspanins CD9 and CD81. CD36 inclusion in this complex facilitates its association with ITAM-bearing adaptor Fcγ receptors (FcγR), allowing CD36-dependent Syk activation and the internalization of ligand-bound CD36 (47) (Figure 1A; Table 1). In addition, CD9 functionally associates with FcγRs, modulating signals for phagocytosis, and FcγR-mediated immune responses (Table 1). Cross-linking of CD9-FcγRIII induces colocalization of CD9, αMβ2 integrin and F-actin, promoting macrophage activation (48) (Figure 1A). In human monocytes and skin-derived DCs, CD9 and CD81 are molecular partners of the trimeric form of FcϵRI (Figure 1; Table 1), the high-affinity receptor for IgE, and are overexpressed in patients with atopic dermatitis (49).

The tetraspanin CD81 plays an important role in Ag-induced B cell activation, B cell development, and survival. It associates functionally with CD19 and CD21, which are members of the BCR complex (3, 54) (Table 1). CD81 deficiency in humans and mice leads to antibody deficiency syndrome by preventing CD19 surface expression (21, 63). Moreover, visualization of primary B cells by super-resolution microscopy shows that CD81-enriched microdomains and the actin cytoskeleton regulate CD19 mobility and organize CD19 and BCR interactions, controlling BCR downstream signaling (64).

In the context of viral infection, CD81 was identified as a receptor for hepatitis C virus (HCV) (65), not only in hepatocytes but also in B cells, T cells, NK cells, and DCs (66). The dynamic properties of CD81 at the membrane are essential for HCV infection (67). Anti-CD81-specific antibodies mediate protection against HCV infection *in vivo*, further demonstrating the functional consequences of this recognition (68). Tetraspanin dynamics at the membrane are also exploited by other viruses. For example, CD9 and CD81 negatively regulate human immunodeficiency virus 1 (HIV-1)-induced membrane fusion (69).

### TEMs during Antigen Processing and Presentation

**Figure 2:**
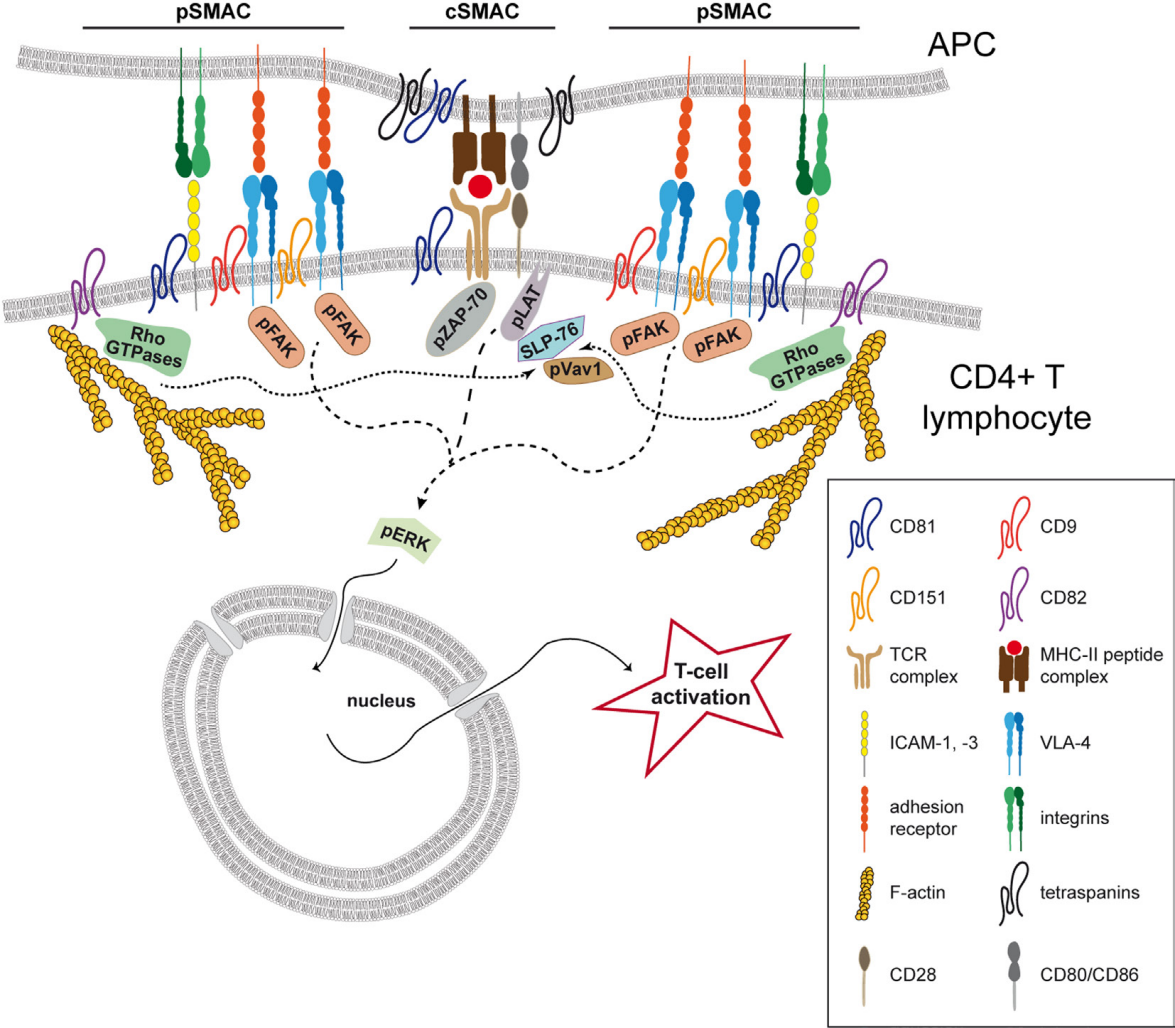
**Tetraspanins organize the T-cell immunological synapse**. Tetraspanin CD81 regulates the organization of the immunological synapse (IS) in CD4^+^ T lymphocytes through the association with CD3ζ at the central SMAC (cSMAC). CD81 controls the localization of the TCR complex and its downstream signaling, positively modulating the phosphorylation of ZAP-70, LAT, and ERK1/2 (dashed line). At the peripheral area of the cell–cell contact (pSMAC), tetraspanins CD9, and CD151 are important for integrin VLA-4 relocalization and activation, positively regulating the integrin downstream phosphorylation of FAK and ERK1/2 (lines with small dashes). At this location, CD81 also interacts with the adhesion receptor ICAM-1, regulating its segregation during IS maturation. Tetraspanin CD82 accumulates at the pSMAC and triggers actin polymerization and the activation of the Rho GTPase pathway (RhoA, Rac1, and Cdc42). The activation of this pathway induces the phosphorylation and the association of Vav1 and SLP76 (dotted lines), potentiating the phosphorylation of the TCR signaling molecules LAT and ZAP-70. In APCs, CD81 is enriched at the IS and several tetraspanins are described to associate with MHC-II. Moreover, CD151, CD37, and Tssc6 were described to regulate antigen presentation by DCs.

T cell recognition of specific antigenic peptides bound to MHC-I and MHC-II molecules on DCs leads to T cell activation and subsequent initiation of T cell-mediated immune responses. In DCs, mechanisms regulating MHC-II intracellular transport are well known (70), and tetraspanins have a role in this process since several tetraspanin family members associate with MHC-II molecules. Interactions between MHC-I molecules and tetraspanins CD53, CD81, and CD82 have been described (71) (Figure 1C). Moreover, tetraspanins CD9, CD81, CD82, CD63, CD53, and CD37 interact with MCH-II molecules (50, 55, 57, 72) (Figures 1C and 2; Table 1). These interactions might lead to the regulation of MHC-II subcellular distribution. CD9, CD53, and CD81 associate with MHC-II at the plasma membrane (50) (Figure 1C; Table 1). In contrast, CD82 and CD63 are highly enriched in MHC-II-enriched compartments (MIIC) (Figure 1B; Table 1), particularly in intraluminal vesicles, where they associate with each other and with the chaperone HLA-DM, playing an important role in the late stages of MHC-II maturation (50, 57) (Table 1). Analysis of protein dynamics by Föster resonance energy transfer (FRET) in MIIC shows that CD63 stably associates with MHC-II and regulates MHC-II surface expression, whereas CD82 associates with HLA-DM without affecting MHC-II expression (58). Knockdown of CD63, CD82, CD9, or CD81 did not prevent MHC-II peptide loading (58). In addition, live cell imaging studies have shown differential CD63 and CD82 subcellular localization in the context of DC phagocytosis. Whereas CD63 and MHC-II are specifically recruited to yeast-containing phagosomes after phagosomal acidification (43), CD82 and MHC-II molecules are recruited to fungal and bacterial phagosomes before fusion with lysosomes and phagosomal acidification (44) (Figure 1B; Table 1). These results support a role for CD63 and CD82 in the dynamic intracellular trafficking of MHC-II after pathogen uptake, playing non-redundant roles in these processes.

Tetraspanins are also involved in the clustering of MHC molecules (Figure 1C). APCs express very small amounts of relevant MHC-II–peptide complexes on the plasma membrane. These MHC-II–peptide complexes are organized and clustered on the cell surface, allowing efficient cross-linking of TCRs and promoting Ag-specific T cell activation (73). It is widely accepted that MHC-II molecules are concentrated into two types of membrane microdomains, TEMs, and lipid rafts (74). The composition and dynamics of these microdomains are essential factors in the outcome of T cell activation. Evidence from a model of raft disruption in B cells suggests that MHC-II association with lipid rafts is important for presentation of Ag at low concentrations (75). Other studies report that TEMs contain MHC-II molecules loaded with a restricted antigenic peptide repertoire, together with HLA-DM and the costimulatory molecule CD86. In contrast, raft-associated MHC-II molecules display a highly diverse set of peptides (51) (Table 1). However, these results are controversial, since the MHC-II determinant CDw78, which is used to identify selectively tetraspanin-associated MHC-II, also defines a conformation of peptide-bound MHC-II acquired through the trafficking to lysosomal compartments (76). Moreover, TEM-induced MHC-II clustering is also supported by evidence that CD9 is required to facilitate the formation of I-A/I-E MHC-II multimers, which are responsible for enhancing the T cell stimulatory capacity of DCs (52) (Table 1). However, a subsequent study showed that cholesterol depletion disrupts MHC-II I-A/I-E interactions, whereas the absence of CD9 or CD81 has no effect (77). This controversy might be due to the differential sensitivity of microdomains to cholesterol depletion. Although TEMs are more resistant to cholesterol depletion than lipid rafts, partial disruption is also observed under certain conditions. Therefore, it is possible that rafts and TEMs both contribute to MHC clustering.

Studies derived from tetraspanin-deficient mice have shown that certain tetraspanin members do not promote MHC multimerization, being rather involved in Ag presentation. DCs from CD37^−/−^ or CD151^−/−^ mice induce hyperstimulation of T cells (56), and similar results were obtained with DCs from Tssc6^−/−^ mice and CD37^−/−^ Tssc6^−/−^ double knockout mice (78). CD37^−/−^ DCs induce T cell hyperstimulation through a mechanism that regulates MHC-dependent Ag presentation, whereas CD151 in DCs regulates T cell costimulation (56) (Figures 1C and 2; Table 2). DC maturation is required for effective T-cell costimulation and involves the upregulation of costimulatory and adhesion molecules (79, 80). In contrast to conventional DCs, plasmacytoid DCs lack CD9 surface expression, which could be responsible for their significant low expression of MHC-II and limited T cell stimulatory potential (80). TEMs thus play a well-documented role in the regulation of different aspects of the MHC-II lifecycle in APCs, including MHC-II clustering and intracellular trafficking of peptide–MHC-II complexes to the APC plasma membrane.

**Table 2:** **Tetraspanin associations in T cells and their role at the immunological synapse**.

Tetraspanin	Associated proteins	Signaling pathway	Function	Reference
CD81	CD3ζ	ZAP-70, LAT, ERK1/2	Controls TCR relocalization to the IS and subsequent downstream signaling	(81)
	CD3δ, CD4, CD8		Association at the membrane	(82–84)
	VLA-4		Association at the membrane	(85)
	ICAM-1		Regulates ICAM-1 distribution at the IS	(81)
CD9	VLA-4	FAK, ERK1/2	Mediates VLA-4 accumulation at the IS and integrin downstream signaling	(86)
	LFA-1		Controls LFA-1-dependent adhesion	(28)
CD151	VLA-4	FAK, ERK1/2	Regulates VLA-4 accumulation at the IS and integrin downstream signaling	(86)
CD82	Actin	Rho GTPases, Vav1, and SLP76	Is enriched at the IS, regulating actin polymerization and TCR downstream signaling	(87–89)
	VLA-4		Association at the membrane	(85)
	CD4, CD8		Association at the membrane	(82–84)
CD53	VLA-4		Association at the membrane	(85)
	CD2		Association at the membrane	(90)
CD63	VLA-4		Association at the membrane	(85)

## Role of Tetraspanins in the Organization of T-Cell Immunological Synapses

### The Immunological Synapse

The initiation of T cell activation mediated by APCs, mainly DCs, requires the establishment of a dynamic structure formed at the cell–cell contact called the immunological synapse (IS) (Figure 2). This structure is characterized by a dynamic spatiotemporal recruitment of Ag receptors, costimulatory molecules, and adhesion proteins to specific zones at the T cell–APC interface. At the T cell side of mature IS, TCR microclusters are clustered together with costimulatory proteins, signaling molecules, and other signaling adaptors at the central supramolecular activation complex (cSMAC) (91–96). More specifically, preexisting TCR nanoclusters (97) concatenate into microclusters, as demonstrated with high-resolution imaging techniques like photoactivated localization microscopy (PALM) and stimulated emission depletion (STED) (98–100). These microclusters form in the periphery of the IS and are translocated toward the cSMAC in a process dependent on the actin cytoskeleton (93, 94, 98, 101, 102). The central area is surrounded by a peripheral SMAC (pSMAC), where integrins and adhesion receptors are localized (81, 91, 102–104). The super-resolution optical techniques near-field scanning optical microscopy (NSOM) and single-dye tracking (SDT) revealed that, like the TCR, LFA-1 is preorganized into nanoclusters that coalesce into microclusters after ligand binding (105, 106). The stability of the IS depends on the binding of integrins, not only lymphocyte function-associated antigen 1 (LFA-1; αLβ2) but also very late antigen 4 (VLA-4; α4β1), to their ligands, the adhesion receptors intercellular adhesion molecule-1, -3 (ICAM) in the case of LFA-1 (91, 103, 104, 107–109). The VLA-4 ligand at the T-cell–APC interface remains unknown (104). In resting T lymphocytes, integrins are mostly in an inactive bent conformation, with low affinity and avidity for ligands. TCR stimulation triggers intracellular signaling that leads integrins to adopt an intermediate-affinity conformation, and then the extended high-affinity conformation (110). These conformational changes induced by TCR signaling modify integrin avidity through a process called inside-out signaling (111), which ultimately regulates integrin affinity for their ligands (112, 113). LFA-1 engagement by its ligand ICAM-1 triggers outside-in signaling, inducing cytoskeletal reorganization that recruits T cell signaling proteins to the IS (113–115).

Both the TCR and LFA-1 modulate cytoskeletal dynamics. TCR signaling triggers actin polymerization enabling the extension of the actin network downstream of LFA-1 (116). VLA-4 costimulation regulates the cytoskeletal movements that drive TCR microclusters associated with signaling complexes to the central area of the IS (109). Moreover, it has been suggested that TCR microcluster formation is dependent on actin polymerization (94, 101); however, other investigators claim that actin is necessary only for microcluster maintenance (98). Continuous actin retrograde flow sustains T cell signaling and signal termination at the central area of the IS (94, 101, 102, 117). Actin filaments are also important for the segregation of adhesion molecules to the pSMAC (91, 102, 103), and actin centripetal flow is essential for the maintenance of LFA-1 in a high-affinity conformation at this location (118, 119).

The T cell cytoskeletal network thus plays an essential role in the spatial organization of the IS. However, the precise mechanisms by which molecules are specifically partitioned into central and peripheral areas of the IS remain an open question. It has been proposed that this segregation is supported by size differences in the ectodomains of immune surface interacting proteins; e.g., LFA-1-ICAM-1 (40 nm) and CD2-CD58 (15 nm) (120–122). In agreement with this view, evidence suggests size-dependent exclusion from the cSMAC of large phosphatases such as CD45, thus allowing the initiation of TCR signaling (94, 123). Recent data show that CD45 is already excluded from preexisting TCR microclusters (124). Given that the TCR in naïve T cells is already clustered with signaling molecules, and that numerous proteins that are translocated to, rearranged and accumulated at the IS are known to associate with tetraspanins, we postulate that protein–protein interactions driven by TEMs actively contribute to IS architectural organization.

### Tetraspanins and the Distribution of Receptors at the T-cell IS

Tetraspanin CD81 accumulates at the IS in both T lymphocytes and APCs (125) (Figure 2), and we recently found that CD81 is an important molecular organizer of the IS structure at the T cell side (81). Fluorescence recovery after photobleaching (FRAP) experiments indicate that CD81 is mostly confined to the cSMAC in the early IS (81), where it colocalizes with the CD3ζ component of the TCR complex (81, 125) (Figure 2). Analyses by phasor fluorescence-lifetime imaging microscopy (phasorFLIM)-FRET reveal that CD81 associates with CD3ζ at the cSMAC of the early IS (81) (Figure 2). In the late IS, CD81 and CD3ζ spread throughout the cell–cell contact and CD81 diffusion decreases, suggesting stable protein–protein interactions throughout the IS. In agreement with this view, CD81 and CD3ζ interaction increases with the IS maturation (81). As a molecular organizer, CD81 controls CD3ζ relocalization to the cSMAC, and the efficient maintenance of the CD3 signaling complex at the cell–cell contact (Figure 2). Hence, CD81 knockdown reduces the number of CD3ζ microclusters at the cSMAC, as detected by total internal reflection microscopy (TIRFM), and impairs TCR downstream signaling, reducing the phosphorylation of CD3ζ, ZAP-70, LAT, and ERK1/2 (81) (Figure 2; Table 2). Moreover, pretreatment of T cells with soluble GST-LEL-CD81 (81), which decreases membrane diffusion of the protein (33), increases T cell activation (81), further indicating that CD81 regulates T cell activation by controlling the duration of TCR signaling at the membrane. A direct CD81-mediated signaling does not seem to be involved in this process, since CD81 C-terminal peptides do not affect T cell activation (81). Thus, by organizing TEMs CD81 regulates spatial molecular organization during the maturation of the IS.

In T lymphocytes, different tetraspanins associate with receptors that are enriched at the IS. In addition to CD3ζ (81), CD81 also interacts with the CD3δ subunit of the TCR complex (84) (Table 2). CD9 localizes with TCR signaling molecules in lipid microdomains (10), CD81 and CD82 associate with CD4 and CD8 coreceptors (82, 83) (Table 2), and CD53 interacts with the costimulatory receptor CD2 (90) (Table 2). It is therefore conceivable that the IS architectural organization of these receptors depends on their inclusion in TEMs through interaction with different tetraspanins. Further research is required to address this notion.

### Adhesion Molecules, Tetraspanins, and the Stabilization of the T-cell IS

Integrins and adhesion receptors are also included in TEMs. In T cells, CD9 interact with LFA-1 (28), CD81, CD82, and CD53 with VLA-4 (85), and CD81 with ICAM-1 (81) (Table 2). In the immune system, tetraspanins regulate cell–cell adhesion through LFA-1 and ICAM-1: CD81 and CD82 promote T-APC cell–cell interaction (126, 127); CD81 induces thymocyte aggregation (128); and CD53 modulates NK and B cell aggregation (129, 130). Conversely, leukocyte LFA-1-dependent adhesion is negatively regulated by CD9 (28) (Table 2). Integrin adhesiveness can be regulated by several mechanisms, such as alterations in the affinity of individual integrin molecules or changes in their clustering on the cell surface or their interactions with ligands. Tetraspanins can modulate integrin activity through various mechanisms. For example, CD81 modulates VLA-4 avidity for its ligand VCAM-1, and CD151 stabilizes α3β1 integrin in its active conformation and regulates α6 integrin diffusion at the plasma membrane (27, 131, 132). CD9 promotes β1 activation, LFA-1 aggregation, and in leukocytes it seems to be essential for a balanced regulation of β1 and β2 integrin activity: it increases β1 adhesion to fibronectin but diminishes LFA-1-mediated adhesion (28, 133).

At the IS, CD81 regulates pSMAC organization through association with the adhesion receptor ICAM-1, controlling ICAM-1 segregation at the cell–cell contact during IS maturation (81) (Figure 2; Table 2). CD81 knockdown decreases the proportion of early synapses, in which ICAM-1 is confined to the pSMAC, and increases the proportion of late synapses (81). During maturation of the IS, ICAM-1 redistributes throughout the entire cell–cell contact, with increasing colocalization and molecular interaction with CD81 (81). T cell activation is also regulated by other tetraspanins. CD9 and CD151 modulate VLA-4 ­accumulation at the IS (86) (Figure 2; Table 2). Interestingly, the IS enrichment of β1 integrins in a high-affinity conformation is impaired in T cells knocked-down for CD9 and CD151, suggesting that integrin activation upon IS formation occurs within TEMs (86). The conformational changes of β integrin extracellular domains can be controlled by the actin linker protein talin (134), which accumulates at the pSMAC (91) and is required for LFA-1 activation mediated by the TCR (135). However, CD9 and CD151 knockdown does not alter talin relocalization to the IS, indicating that these tetraspanins are not involved in the regulation of integrin inside-out signaling (86). Integrins and adhesion molecules can act as signaling receptors. Integrin or ICAM-1 costimulation triggers T cell activation (136–138), and LFA-1 coengagement with the TCR lowers the T cell activation threshold (139, 140). VLA-4 ligation also costimulates T cells in a TCR-dependent manner (141), and polarizes T lymphocytes toward Th1 responses (104). LFA-1 and VLA-4 activation is controlled by the interaction with a cascade of adaptor and signaling proteins (142, 143), and these downstream signaling can be modulated by tetraspanins. CD151 supports the phosphorylation of FAK, Src, and p130CAS (144) and promotes the activation of small GTPases and ERK1/2 in an integrin-dependent manner (145, 146). ERK1/2 signaling is also increased by CD9 (147). During T-APC cognate cell–cell interactions, CD9 and CD151 knockdown reduces FAK and ERK1/2 phosphorylation, and impairs the enrichment of phosphorylated FAK at the IS (86) (Figure 2; Table 2). Tetraspanins CD9 and CD151 are therefore important for integrin enrichment at the IS, modulating integrin downstream signaling.

As previously mentioned, the actin cytoskeleton plays a crucial role in the regulation of the spatial organization of TCRs and adhesion molecules at the IS. The links between tetraspanins, membrane receptors, adhesion proteins, and the actin cytoskeleton suggest a possible regulation of this process by TEMs. CD81 and CD9 are connected to the actin cytoskeleton through α-actinin and ezrin-radixin-moesin (ERM) proteins (148, 149). CD151, CD81, and CD82 regulate the actin cytoskeleton through RhoA and Rac1 signaling molecules (62, 150–152). In T lymphocytes, CD82 costimulation triggers actin polymerization and T-cell activation by stabilizing signaling downstream of TCR/CD3 (87, 88) (Figure 2; Table 2). T cell morphological changes induced by CD82 engagement depend on the activity of Rho GTPases (RhoA, Rac1, and Cdc42), involving the association of Vav1 and the adapter molecule SLP76 with the Rho GTPase pathway (88). Importantly, CD82 is enriched at the IS in an actin-dependent manner (89) (Figure 2; Table 2). CD82-dependent regulation of the actin cytoskeleton during T cell activation may involve its interaction with LFA-1. CD82 regulates T cell-APC adhesion-dependent signaling (153), through its interaction with LFA-1 (126), and like LFA-1, CD82 localizes at the pSMAC (89) (Figure 2). At the IS, CD82 seems to stabilize interactions with the actin cytoskeleton, favoring the formation of signaling complexes. It would be interesting to determine whether CD82 dynamics depend on its association with LFA-1, and whether CD82 can modulate LFA-1 functions.

Thus, at the IS, tetraspanins CD9, CD81, CD82, and CD151 mediate the connections between adhesion molecules, the actin cytoskeleton and signaling complexes. Increasing evidence highlights the importance of TEMs in the organization of the temporal and spatial molecular distribution at the IS, generating the context that allows full T cell activation.

## Concluding Remarks

In APCs, different receptors involved in pathogen recognition and Ag presentation are associated with tetraspanins. Further investigations are necessary to determine the spatial distribution and segregation of receptors within TEMs, as well as the importance of these microdomains in the regulatory mechanisms of receptor functions and downstream signaling. The establishment of long-lasting T cell–APC contacts, which lead to the formation of the IS and ultimately promote an efficient T cell activation, are required for the initiation of T cell-mediated immune responses. IS stability depends on the binding of integrins to adhesion receptors upon TCR ligation, triggering downstream signaling. The complex IS architectural organization depends on the inclusion of the receptors concentrated at the IS into TEMs, through their dynamic and spatiotemporal interactions with different tetraspanins. The important role of TEMs in the regulation of the dynamic process of IS formation has been recently emphasized. These specialized membrane domains allow the compartmentalization of receptors and adhesion molecules and connect them to the cytoskeleton and signaling complexes that induce T cell activation. The development of advanced microscopy techniques will provide further insight into IS dynamics and the contribution of TEMs and other microdomains to this process. Considering the plasticity of the interactions that take place in TEMs, strategies that regulate IS organization by targeting tetraspanins could allow therapeutic manipulation of the final outcome of T cell activation and the subsequent immune response.

## Author Contributions

VR-P, FS-M, and GMH had scientific discussion for this work and wrote the manuscript.

## Conflict of Interest Statement

The authors declare that the research was conducted in the absence of any commercial or financial relationships that could be construed as a potential conflict of interest.
